# Experiences of Alaska Native people living with burn injury and opportunities for health system strengthening

**DOI:** 10.1186/s12913-023-10243-x

**Published:** 2023-11-15

**Authors:** Mallory B. Smith, Elisha Brownson, Andrea K. Newman, Christopher Madison, Molly Fuentes, Dagmar Amtmann, Gretchen J. Carrougher, Nicole S. Gibran, Barclay T. Stewart

**Affiliations:** 1 Harborview Injury Prevention & Research Center, University of Washington, 325 9th Ave, Box 359796, Seattle, WA 98104 USA; 2 Division of Pediatric Critical Care Medicine, Department of Pediatrics, University of Washington, Seattle, WA USA; 3 Department of Surgery, Alaska Native Medical Center, Anchorage, AK USA; 4 Department of Rehabilitation Medicine, University of Washington, Seattle, WA USA; 5 Native Village of Kotzebue, Kotzebue, AK USA; 6 Division of Trauma, Critical Care, and Burn, Department of Surgery, University of Washington, Seattle, WA USA

**Keywords:** Burn injury, Alaska, Community reintegration, Rehabilitation

## Abstract

**Background:**

Injuries are a leading cause of death and disability for Alaska Native (AN) people. Alaska Native Tribal Health Consortium (ANTHC) is supporting the development of a burn care system that includes a partnership between Alaska Native Medical Center (ANMC) in Anchorage, AK and UW Medicine Regional Burn Center at Harborview Medical Center (HMC) in Seattle, WA. We aimed to better understand the experiences of AN people with burn injuries across the care continuum to aid development of culturally appropriate care regionalization.

**Methods:**

We performed focus groups with twelve AN people with burn injury and their caregivers. A multidisciplinary team of burn care providers, qualitative research experts, AN care coordinator, and AN cultural liaison led focus groups to elicit experiences across the burn care continuum. Transcripts were analyzed using a phenomenological approach and inductive coding to understand how AN people and families navigated the medical and community systems for burn care and areas for improvement.

**Results:**

Three themes were identified: 1-Challenges with local burn care in remote communities including limited first aid, triage, pain management, and wound care, as well as long-distance transport to definitive care; 2-Divergence between cultural values and medical practices that generated mistrust in the medical system, isolation from their support systems, and recovery goals that were not aligned with their needs; 3-Difficulty accessing emotional health support and a survivor community that could empower their resilience.

**Conclusion:**

Participants reported modifiable barriers to culturally competent treatment for burn injuries among AN people. The findings can inform initiatives that leverage existing resources, including expansion of the Extension for Community Healthcare Outcomes (ECHO) telementoring program, promulgation of the Phoenix Society Survivors Offering Assistance in Recovery (SOAR) to AK, coordination of regionalized care to reduce time away from AK and provide more comfortable community reintegration, and define rehabilitation goals in terms that align with personal goals and subsistence lifestyle skills. Long-distance transport times are non-modifiable, but better pre-hospital care could be achieved by harnessing existing telehealth services and adapting principles of prolonged field care to allow for triage, initial care, and resuscitation in remote environments.

**Supplementary Information:**

The online version contains supplementary material available at 10.1186/s12913-023-10243-x.

## Introduction

Injuries are a significant problem in American Indian and Alaska Native (AI/AN) communities related to more dense exposures to hazards, a disproportionately high incidence, and limited access to definitive trauma and rehabilitative care [1]. As the leading cause of death for AI/AN individuals between the ages of 1 to 44 and the third leading cause of death for all ages, there is a substantial disparity with regard to the burden of injury between the AI/AN population and other racial/ethnic groups in the United States (US) [2, 3]. In addition, the AI/AN death rate due to fire and smoke injury is more than twice that of all other racial/ethnic groups in the US [2, 3]. The potential health, social and financial burdens associated with injuries exacerbate the already significant health disparities resulting from historical traumas, including colonization, systematic genocide, forced relocation, and boarding school placements and differential access to economic opportunities and essential health services [4–6].

Burn injuries frequently result in life-long sequelae such as scarring, changes in outward appearance and body image, amputations, and symptoms of posttraumatic stress that can reduce survivors’ social, mental and physical functioning [7–9]. Organized acute burn care, coordinated multidisciplinary follow-up, and structured rehabilitation programs are necessary to prevent, identify and/or manage these potential sequelae and maximize community functioning for people living with burn injury. Recent work has identified certain individuals who may be at higher risk for unsatisfactory long-term outcomes including individuals who live far away from multidisciplinary burn centers, have barriers to transportation, are experiencing homelessness, and/or who have substance use disorders [10, 11].

Alaska has two Level II adult/pediatric trauma centers, both located in Anchorage. There is no verified burn center in the state. The regional burn center for Alaska is the UW Medicine Regional Burn Center at Harborview Medical Center in Seattle, WA. Transport distances can be 800 miles or more to get to Anchorage; the flight distance from Anchorage to Seattle is 1,445 miles. Prior to 2016, most complex burn care for AN people was managed by the multidisciplinary team at the UW Medicine Regional Burn Center at Harborview Medical Center in Seattle, WA. In 2016, Alaska Native Tribal Health Consortium (ANTHC) supported a burn surgeon and development of a burn care system based in Anchorage, AK at Alaska Native Medical Center (ANMC) (e.g., education of multidisciplinary stakeholders, strategic planning, care coordination, outreach initiatives). By doing so, ANMC achieved the capacity to expertly care for burn-injured patients closer to home and with more culturally appropriate care (Table 1). Additionally, ANTHC supported community health worker education, tele-education programs for providers who might care for burn-injured patients statewide and formalized a collaborative relationship between ANMC and UW Medicine Regional Burn Center for patients who require complex and/or longitudinal care.

**Table 1:** Characteristics of Alaska Native Medical Center and Harborview Medical Center/UW Medicine Regional Burn Center

	Alaska Native Medical Center	UW Medicine Regional Burn Center at Harborview Medical Center
Mission population	Alaska Native people; people who require specialized emergency, trauma or burn care	Limited English proficiency; uninsured/underinsured; people incarcerated in King County’s jails; people with mental illness or substance abuse problems; people who require specialized emergency, trauma or burn care
Trauma center designation	Adult and Pediatric Level II	Adult and Pediatric Level I
Total beds	173	413
Burn/flex intensive care beds	22 adult ICU beds, 4 PICU beds (no designated burn beds)	18 adult or pediatric ICU beds
Burn/flex inpatient beds		23
Burn Admissions per year	50	800
Burn surgeons	1	4

ICU – intensive care unit, PICU – pediatric intensive care unit

The Alaska burn care capacity development has been a success story; however, there is more to do. Given resource limitations, it is important that ongoing health system strengthening initiatives are tailored to meet patients’ needs and are designed and executed with people living with burn injury as collaborators. Therefore, we sought to better understand the experiences of AN people with burn injuries throughout the care continuum as burn care capacity in Alaska were expanding and sought their guidance on future health system strengthening initiatives.

## Methods

### Study design and setting

We conducted focus groups of burn injured AN people and their carers (e.g., parents or guardians) utilizing a semi-structured interview. Focus groups took place in Anchorage, Alaska at Alaska Native Medical Center and occurred over two days. The transcripts were analyzed using a phenomenological approach to understand the experiences of navigating the medical and community systems for burn care.

### Participants and sampling

Participants were either individuals who received care for a burn injury or parents of children who received care for a burn injury at ANMC in Anchorage, Alaska and/or UW Medicine Regional Burn Center at Harborview Medical Center (HMC) in Seattle, Washington. Participants were identified through convenience sampling due to their contacts with the burn teams, and particularly those known to have complex injuries, challenging recoveries, and/or live in remote communities.

### Ethics approval and consent to participate

#### Ethical approval

for this study was granted to the Burn Model System National Data and Statistical Center by University of Washington Human Subjects Division (Study ID# 00001529). Participants were approached via telephone and after clinic visits. No one refused to participate. All participants provided informed consent before taking part in the focus groups, being recorded, and having the findings published. Costs of travel and accommodation were not borne by participants and travel to and stay in Anchorage were aligned with scheduled clinic visits. The focus groups took place at Alaska Native Medical Center. Participants were given the option to bring one or two members of their support system to make the more comfortable. Participants received light lunch as compensation for their time during the focus groups. All methods were carried out in accordance with relevant guidelines and regulations. All authors and Alaska Native Tribal Health Consortium (ANTHC) approved the manuscript for publication.

### Interview process

The interview guide was developed to elicit information about participants’ experiences at multiple timepoints spanning the burn care continuum (e.g., first aid and prehospital care, acute care and inpatient rehabilitation, transition to Alaska and their communities, community-based rehabilitation, return to work and/or subsistence living and/or their roles prior to injury) (Supplementary Material). The discussions were conversational and led by open-ended questions to gain more understanding of the events that took place, how the participants felt, what they experienced, and what they thought could have been better. The focus groups were led by a multidisciplinary team of Alaskan and non-Alaskan burn care providers that included an expert in qualitative research, nurse coordinators with extensive focus group experience, burn care specialists, and a cultural liaison. Participants knew one or two of the interviewers but not all five of them. No repeat interviews were performed.

### Qualitative analysis

Audio transcripts were analyzed using an inductive and deductive phenomenological approach (Hsieh, 2005). Field notes were reviewed alongside the transcripts to aid coding. Transcripts were not reviewed by the participants. The primary coding team consisted of one physician (MS), one rehabilitation psychologist (AN), and one rehabilitation medicine physician (MF) with qualitative and burn research experience. Codes were derived from a close reading of the transcripts to capture key concepts. Codes were then grouped into themes based on how they were related to each other or the phase of burn care they pertained to [12]. Two coders reviewed the transcripts in their entirety using Atlas.ti. The coding team met regularly to discuss the emerging themes and areas of disagreement in the codes. Disagreements were arbitrated by a third coder when needed. Once an initial set of themes were identified, an Alaska Native member of the focus groups (CM) and burn care specialists from ANMC (EB) and UW Medicine Regional Burn Center (BS, GC) who participated in the focus groups served as member checks to validate the findings. The consolidated criteria for reporting qualitative studies (COREQ) was used to design, describe, and report the study.

## Results

**Fig. 1:**
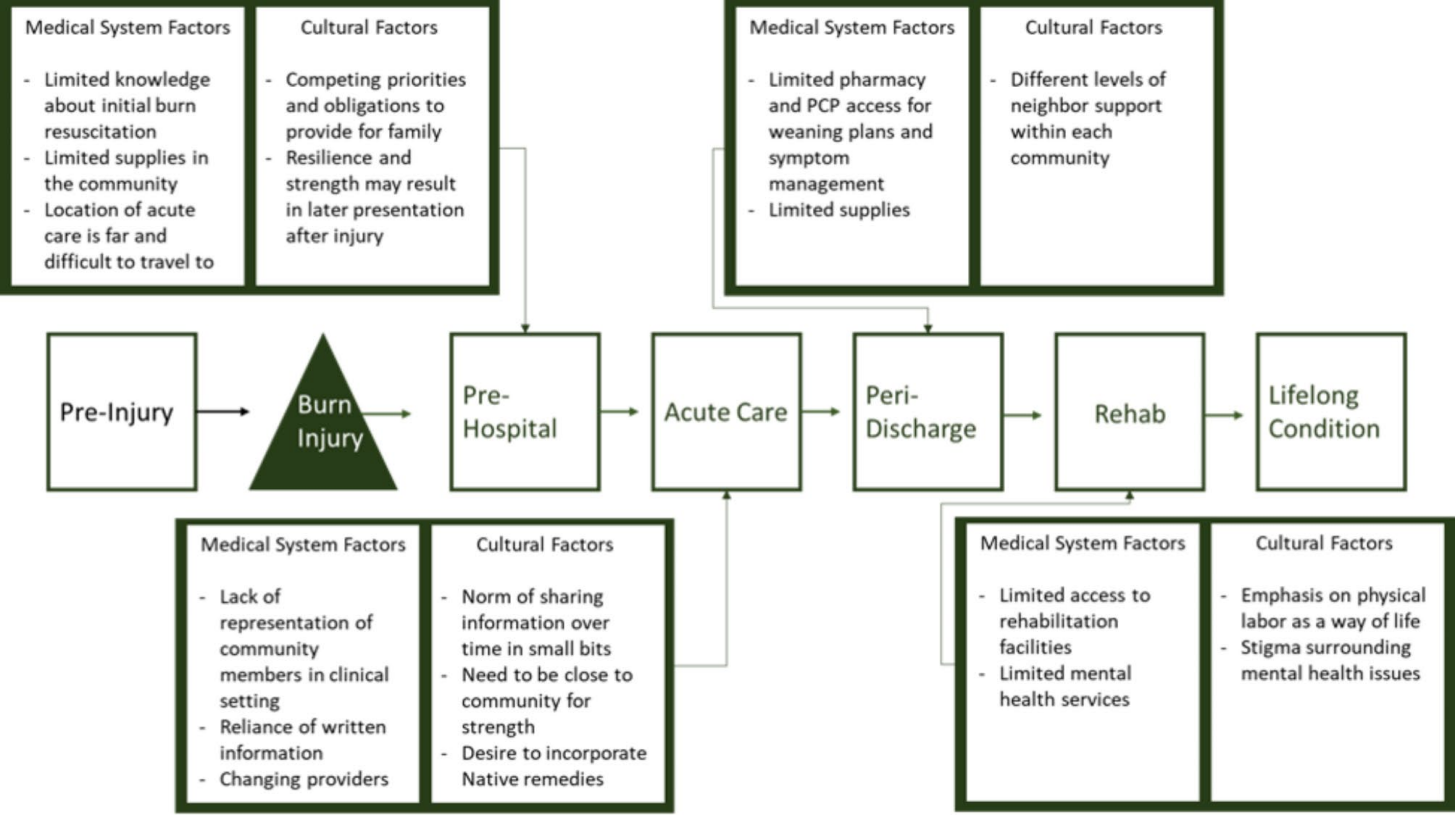
Identified factors along the burn care recovery continuum that contribute to health disparities for Alaska Native people living with burn injuries

There were twelve total participants (ages 24–61, seven were female, three were carers of children with burn injury). Injuries ranged from deep palmar contact burns in a child who received predominantly outpatient care to an adult with 80% total body surface area burn. All participants experienced some form of long-term impairment. Participants represented those from both urban and rural/remote Alaska and care provided within the state and in both Washington and Alaska. The extremes of travel were well represented with those who could drive within Anchorage for burn care and those who traveled via snow machines, boats, small planes (e.g., village to Anchorage), and large planes (e.g., Anchorage to Seattle). We analyzed 5.42 h of recorded transcripts. Three main themes emerged from the data related to the experience of receiving care for burn injuries by AN people: [1] challenges with local burn care; [2] mismatch of cultural values and medical practices; and [3] challenges accessing emotional health support (Table 2). As the themes emerged, components within each theme were charted onto a timeline of burn care that highlighted the medical system and cultural factors at play resulting in the experiences and feelings of the participants (Fig. 1).

**Table 2:** Additional quotes related to themes identified by Alaska Native people with burn injury and/or their carers provided in focus groups

Themes	Phase of Care	Quotes
**Challenges with local burn care**	Pre-hospital	“So there’s a delay in getting help.” “But they have to travel maybe 50 miles, just about 50 miles or 90 miles.”
	Transitions of care	“…ran out of Xeroform [gauze], too, for a while. We had to improvise with the smaller ones.” “It was hard. Especially [because] I didn’t have lotion, because I couldn’t use smelly lotion because it would break out. I tried to get it from the pharmacy but they said I’d get it from the clinic, but I didn’t.”
**Mismatch of cultural values and medical practices**	Acute care	“Someone you can trust and someone who you know personally was taking care of you before…that holds a lot more weight than a stranger.” “…was missing (traditional) food (choices) from home.”
	Transitions of care	“I don’t expect the prescribed creams to help.” “I’m a visual person. Pictures are good.”
	Rehabilitation	“Maybe if there was some way where you could construct talking about hunting season and fishing season…that’s how we think of the year.” “People would understand better [a question] of are you able to sit on a snowmachine before winter.”
**Challenges accessing emotional health support**	Rehabilitation	“I didn’t know who else to talk to. I wasn’t prepared for any of that either. I didn’t think it would be an issue. I was focused on my burdens. So as I got better, I got worse mentality. I got better physically, and I got worse mentally. But I didn’t know who to talk to in the community.”

### Challenges with local burn care

Participants identified challenges in receiving care for their burn injuries in their home villages/towns. These challenges spanned multiple phases of burn care. In the first aid and triage phase, participants reported a lack of local knowledge regarding the appropriate initial steps in resuscitation and pain management. Participants described their attempts to advocate for themselves and their loved ones as well as how they coped with their suffering. One participant described her husband’s initial care:

In the village it’s different. They had him sit in the lobby for 45 min…all his skin was gone, and they had him sitting there, and he was like, “Are you guys going to help me?“…it was bad. I mean, I could smell him, he was still burning…we just sat there waiting.

Participants also described difficulties traveling to higher levels of care after their initial assessment. In many cases, participants had to use a snow machine and/or take multiple flights and spend an entire day traveling to reach definitive care. For the majority of cases, they were provided with a travel escort, often a family member. For parents traveling with their injured child, it was difficult to leave other children behind and to not have their partner or another family member for support. Additionally, participants described being away home for weeks to months while receiving inpatient care, and given many Alaska Natives lead subsistence lifestyles, this resulted in financial burdens on their family.

[The hardest part was] missing my other kids and my husband wanting to go hunting but wasn’t sure who would take care of the game that he caught [because] me and my girls [usually take care of the game].

Participants faced similar difficulties accessing the appropriate care after returning to their home from the burn center. These challenges included limited access to wound care supplies, limited guidance on pain medication weaning, and limited knowledge by local health aids regarding wound care. One mother reported that she took on the responsibility of her child’s wound care due to concerns about the care provided at the local clinic despite insufficient training. Another participant recounted a difficult time weaning off pain medication on her own:

I went back [to my village] to nothing. They didn’t even have gauze. I went through five days of hardship because they didn’t take me off the pain pills. I had to put myself off the pain pills.

### Mismatch of cultural values and medical practices

Important differences in participants’ personal values and the norms established by the medical system were discussed. These differences were most apparent during the acute care phase and around the time of discharge from the index hospitalization and included a mismatch in the following areas: higher level care took place outside of participants’ communities; communication and continuity of care did not always facilitate a relationship of trust; and treatment plans did not include activities of subsistence living or traditional, Native healing methods.

As participants reflected on their experience as an inpatient, they discussed differences between ANMC and HMC. Participants preferred to be admitted to ANMC because it was closer to home and, therefore, easier to access by their families and members of their support systems. One mother described how this proximity made a difference in her daughter’s healing:

Her classmates came to visit while they were in town, and that really uplifted her. After talking with them, maybe an hour later, she let go of the walker and walked.

This family’s experience contrasted to other participant’s stories of being separated from their loved ones without the comforts of home during their admission in Seattle. This separation was viewed so negatively that some participants discussed taking on more responsibilities for their own care in order to avoid having to go to Seattle:

Okay, I don’t want to go to Seattle, so just show me what to do, and I’ll deal with it.

Participants focused on the importance of building trust with the burn care team. Factors that allowed for more trust were repeated encounters with the same healthcare provider(s) and communication styles that aligned with the norms of Alaska Native cultures. Sharing of information done by a provider that had a relationship with the participant was preferred. Additionally, participants preferred printed information to contain pictures or visual descriptions of instructions, which is consistent with the fact that many adult participants expressed a strong preference for information provided visually. When referring to a handout meant to inform the reader about symptoms to expect after discharge and initial steps to take at home, one participant said:

I think one thing that would make me appreciate this more is if you had consistency with your doctor or healthcare provider…knowing that you can trust [she] has [reviewed this], and you trust her, I think I would feel ready to try to make it better.

Participants desired treatment plans that considered the traditional healing methods from their own culture and rehabilitation plans that accounted for their lifestyle and activities of daily living. These themes were specifically prevalent when discussing symptom management for itch, pain, environmental temperature sensitivity/intolerance and scarring. When asked if participants would trust symptom management plans post-discharge more if it was partnered with the local, traditional healers, one participant replied,

Yep. If it incorporated some more local traditions and what you can do [at] home with home remedies.

As other participants discussed what it was like to return to their homes and need to function at their pre-injury baseline, they had discussions about physical labor. Most participants reported that they had help from their neighbors at home but that the level of support varied location to location and diminished over time. While many participants shared stories of generosity from their neighbors after they initially returned, they also described a lack of alternatives/accommodations to doing physical work after their injury. One participant said:

You just do it. Everyday life. You can’t stop. We don’t stop, even if we’re burned. We don’t stop what we do.

Because of this, there was a desire expressed for physical rehabilitation plans to be tailored towards the work involved in their subsistence lifestyles. For example, one participant imagined a physical rehabilitation plan with goals and targets based on fishing-related tasks:

Maybe if you had questions built…are you able to have the hand dexterity to pull in a net by fishing season?

Lastly, in addition to wanting connection to their traditions and lifestyles, participants also reported frequently using their smart phones for communication and education to overcome distance and information gaps. This might suggest that materials and efforts to improve access to care via communication should be optimized for smart phone use. Additionally, interventions for emotional health or peer support should consider leveraging phone use to overcome challenges of distance.

### Challenges accessing emotional health support

The last major theme was based on discussions about “emotional scars” that were more difficult to treat than the physical scars and physical symptoms participants experienced. Their need for emotional support was most evident in their recounts of specific times someone provided understanding and empathy. Many participants could describe in detail the person, place, and words used during this encounter. In most instances, it occurred in an informal setting and not as part of a structured therapy or counseling program. One participant’s story revealed how close he was carrying his emotions and how fresh they still were even though a long time had passed since his injury:

I remember being in the waiting room there was an older lady sitting next to me. She said something along the lines of, “I’m glad to see you out and about. You’ve given us hope.“ I started crying right there.

Outside of these encounters, participants reported difficulties knowing how to process their emotions and how to talk to their families and members of their communities about their emotions and burn injuries. Some individuals reported symptoms of hypervigilance and fear regarding situations that could result in another burn injury. Other individuals reported body image issues and difficulties being seen by and communicating with others. When asked what participants wished they had during their recovery, they reported wanting to know that their emotions were normal for people with burn injuries and to have someone with whom to talk. Specifically, participants expressed the need to talk with other AN people living with burn injuries.

I can’t talk to my husband, and I do need to talk to someone. People who aren’t burned, they don’t understand…you would need to talk to one who went through it.

## Discussion

This study explored the experiences of AN people with burn injuries across the burn care continuum. Although ANTHC and ANMC have made major improvements in burn care capacity across Alaska, AN people with burn injuries continue to face barriers to accessing appropriate care. Participants provided key areas and targets for improved communication, knowledge translation, intervention and evaluation. The findings also highlight the impacts of long-standing underfunding to indigenous health services, the distance, both physical and cultural, between Alaska Native communities and definitive care systems for complex injuries, and the strength and resiliency that AN people have relied on for recovery after injury. Additionally, AI/AN people are underrepresented in burn injury data and intervention planning, which limits the potential benefit of such interventions on the health of excluded individuals. Participants commented on the lack of connection with key outcomes and resources. This extends to standardized measures of health and health system performance that are not well understood or meaningful to AN people due to differences in employment, subsistence living, and community activities. Participants provided insights into how these factors contribute to health disparities for AN people with burn injuries as well as for an agenda to strengthen the regional health system in order to address disparities and generate more culturally competent care.

A recent report found that the largest differences in risk of injury and injury-related mortality occurred for AN people was related to their geography and climate. Compared to White individuals living in Alaska, AN people living in remote areas were more likely to die from land-transport accidents and drowning even if care was reached [13]. This is consistent with our finding that access to adequate first aid, triage and acute care was limited in Alaska Native communities and a barrier to emergency service delivery. The treatment of burns, like submersion injuries and motor vehicle accidents, depends on timely and effective resuscitation with under resuscitation potentially leading to exacerbation of injuries or death and over resuscitation causing preventable morbidities. We found that limited knowledge about initial burn care resuscitation and inadequate supplies for wound care contributed to negative patient experiences. This is likely due, in part, to longstanding underfunding of indigenous health services in the US and not lack of effort by ANTHC or interest among Alaska Native providers working in remote areas [14–16]. Our findings suggest that individuals and communities would be motivated to learn about burn first aid, community-based care measures, and initial burn management. This presents an opportunity to strengthen the local healthcare system with focused education on burn first aid and a modest increase in resources for supplies, protocols, and education. Given this and other feedback, these activities are being prioritized by ANTHC, the Community Health Aide Program, and by Extension for Community Healthcare Outcomes (ECHO) [17]. The Burn and Soft Tissue ECHO Projects is funded by ANTHC and uses videoconferencing technology to connect regional interdisciplinary experts from both ANMC and UW Medicine Regional Burn Center with primary care providers, other health services professionals, and community members across Alaska to facilitate a closer network, education, and improving service delivery. The discussions with, and mentoring from, specialists help equip frontline care stakeholders in their efforts to support individuals and their families with health and disability-related needs in their home communities.

Mental healthcare in Alaska Native communities has been a priority of ANTHC and work to increase the number and scope of community-based mental healthcare workers has resulted in marked improvements in access to care [18]. However, some services within mental healthcare, such as rehabilitation psychology after injury and cognitive processing therapy for people experiencing stress symptoms, remain difficult to access at the community level [19]. Participants described reliance on resilience and a ‘just do it’ work-ethic even when physically, psychologically, and/or socially difficult. Although resiliency and related traits are associated with post-traumatic growth, unmanaged psychological distress can lead to decreased health-related quality of life and inadequate community reintegration. The latter is particularly disconcerting given the importance placed on community and shared responsibilities inherent in subsistence cultures. As guidance, participants reported interest in decentralized mental healthcare services focused on trauma rehabilitation and would readily engage with survivorship groups. Prior work has shown a negative correlation between mental health and cultural engagement and that AN people who felt a connection to their Native culture had lower rates of suicide [20]. Therefore, efforts to facilitate access to culturally competent mental healthcare services and leverage existing peer-support programs (e.g., Phoenix Society SOAR) appear to be logical next steps [21]. There are existing structures using telemedicine for psychiatric treatment and counselling in this population that have demonstrated higher rates of completion of therapy compared to in-person programs. This highlights an opportunity to align with the identified desire to build a community of AN people living with burn injury who can support one another despite being physically apart. Phoenix SOAR connects survivors and loved ones with others who have experienced similar trauma - whether through their own burn injury, or as the loved one of a burn survivor. Empowering Alaska Native survivors, training peer-supporters and initiating regular support groups are similarly achievable goals in the short-term.

Other recommendations that are achievable without significant increases in capacity include improvements to culturally competent care. As example, education and training for burn care team members at UW Medicine Regional Burn Center from AN people with burn injury and experts from ANMC could increase the capacity to provide care in manners more comfortable and appropriate for Alaska Native patients. There is a cultural liaison from ANMC who supports Harborview Medical Center already. Expansion of this program to include incorporation of traditional Alaska Native healing arts and science into acute care and rehabilitation may also be feasible. Similarly, focusing on culturally relevant rehabilitation goals (e.g., dexterity to manage fishing line, strength, and balance to manage a snow machine, adapting to cold sensitivity) could be incorporated into therapy plans and may result in more functional engagement during one of the most challenging times in burn rehabilitation. Further, our findings are aligned with other qualitative studies regarding recovery of people living with burn injury from rural American states, including use of active coping strategies, expressing altruism through helping others, finding meaning and acceptance, and active seeking and use of support [22, 23].

In response to this and other initiatives, the ANTHC and ANMC have taken concrete steps to improve the care of AN people with burn injuries by focusing on increasing the accessibility of mental healthcare, reducing the distance between place of injury and definitive care, and strengthening the existing healthcare structure in remote locations. Several follow-on initiatives are underway or planned: education for health aides on wound care and psychological screening, transfer from HMC to ANMC after intensive and early acute care is complete, development of Alaska-based SOAR supporters and volunteers, reframing of rehabilitation therapy to align with activities of subsistence living, and creating a handoff tool to mitigate the risk of poorly executed transitions of care (Table 3).

**Table 3:** Example needs, action priorities and next steps to strengthen the burn care system for Alaska Native people

Identified Need	Action priorities achieved	Next Potential Steps
Location of acute care	Development of burn care capacity at ANMC	Acute care transition from HMC to ANMC to facilitate stepwise transitions of care
Limited access to mental healthcare post-injury	Support for and empowerment of behavioral therapy aides in remote and rural communities	Facilitate development of an Alaska-specific peer support program that leverages mobile technologies
Need for culturally competent therapies	Integrated into Project ECHO videoconferences	Outdoor therapy and rehabilitation goals tailored to Alaska Native culture (e.g., activities of subsistence living)
Limited long-term follow-up after discharge	Closer and regular follow-up at ANMC for people with burn injuries (e.g., change from 6 month to 3-month long-term follow-ups)	Education of health aides on wound care and psychological screening

ANMC – Alaska Native Medical Center in Anchorage, Alaska; HMC – UW Medicine Regional Burn Center at Harborview Medical Center in Seattle, Washington; Project ECHO – a virtual learning model that uses real time videoconferencing technology to connect a team of interdisciplinary specialists with health and community service professionals, educators, and community members. ANTHC Project ECHO includes Burn and Soft Tissue ECHO.

This study has several important limitations to consider, including selection and recall biases. Recruiting members of the Alaska Native communities to participate in research is limited by a lack of trust in the medical community, distance, and finances to travel, and ability to communicate about the opportunity with all potential members of the target population. The participants in this study had relationships with the burn care team involved in recruitment and were able to travel to Anchorage to participate concurrent with scheduled clinic visits. Future work should focus on understanding the experiences of AN people living with burn injury from a diversity of tribes and communities with differing levels of resources and accessibility. Additionally, for some participants, the focus group took place many months after their injury, which may increase the risk of recall bias. However, our study was not focused on the details of burn care but on the experience of the individuals. It is reasonable that participants remember the way they felt and the important challenges they faced during such a critical time in their lives. Although we had a diverse group of participants with a wide range of burn-related experiences the findings may not be fully generalizable to AN people and other Alaskans more broadly. Lastly, usual risks of bias associated with focus group methodology, power dynamics inherent in research with both patients and clinicians, and cross-cultural communications may have influenced these findings. Regardless, the information provided by the participants represents their real experiences and opportunities for us to improve the continuum of burn are in Alaska.

## Conclusion

ANTHC and ANMC have markedly increased burn care capacity over last decade and should be examined as a success story for working to delivery equitable health services for AN people, while some barriers continue to limit quality health care after burn injury. As examples, AN people with burn injury experience challenges with local burn care in remote communities including limited knowledge regarding steps in first aid, triage and resuscitation, pain management, and wound care, as well as long-distance transport to definitive care. Additionally, there is disparity between cultural values and medical practices that generated mistrust in the medical system, isolation from their support systems, and recovery goals that are not aligned with their needs. Lastly, there remain challenges accessing emotional health and peer support. Efforts like the current one that seek guidance from AN people living with burn injury are critical to defining needs and developing culturally competent health system strengthening initiatives.

### Electronic supplementary material

Below is the link to the electronic supplementary material.

Supplementary Material 1

## Data Availability

The dataset used and/or analyzed during the current study available from the corresponding author on reasonable request.
