# Supplementary material for: Experiences of Alaska Native people living with burn injury and opportunities for health system strengthening
Source: BMC Health Serv Res. 2023 Nov 15;23:1260. doi: 10.1186/s12913-023-10243-x (PMC10652576; doi:10.1186/s12913-023-10243-x)
Supplement: Supplementary file 1 — Supplementary Material 1 [file 12913_2023_10243_MOESM1_ESM.docx]

Alaska Burn Recovery Experience Interview Guide

1. To better understand the barriers to care and recovery for the Native and Non-Native Alaskan burn survivor population,
2. To identify potential research and program development opportunities for improving burn-related recovery, and
3. To identify educational/outreach opportunities in Alaska.

*Please note:*

1. Use open ended questions whenever possible.
2. Be patient - allow participants to discuss what is most important to them.
3. Silence as one gathers their thoughts, is common and should be allowed.

*General guide*

1. Introductions and comfort issues (bathrooms, food and drinks, room temperature)
2. Purpose of these focus groups and what to expect
3. Tell us about your injury, care, and recovery experience.
   1. What did you experience in your community right after you were injured?
   2. Tell us about how you felt when outside of your community or village.
   3. If you received care in Seattle, tell us about how you recovered and accessed what you needed when you returned to Alaska.
   4. What helped you recover in your community?
4. Regarding recovery (i.e., period after hospitalization), what challenges did you face with regard to:
   1. Accessing care
   2. Getting back to your work or community role
   3. Mental health
   4. Physical function
   5. Cultural role
   6. Returning to school
5. What are the specific issues for parents and carers living in Alaska?
